# Geographic variation in clinical outcomes and anticoagulation among medicare beneficiaries with non-valvular atrial fibrillation

**DOI:** 10.1007/s11239-023-02855-1

**Published:** 2023-08-02

**Authors:** Brett D. Atwater, Manuela Di Fusco, Allison Keshishian, Rachel Delinger, Mauricio Ferri, Jenny Jiang, Lauren Seigel, Huseyin Yuce, Jennifer D. Guo

**Affiliations:** 1 Inova Heart and Vascular Institute, Inova Heart and Vascular Institute, 4th floor Medical Directors Suite, 3300 Gallows Road, Falls Church, VA 22042 USA; 2 Bristol-Myers Squibb Company, Lawrenceville, NJ USA; 3 STATinMED, LLC, Dallas, TX USA; 4 New York City College of Technology, City University of New York, New York, NY USA; 5 Pfizer Inc., New York, NY USA

**Keywords:** Atrial fibrillation, Direct oral anticoagulant, Stroke/systemic embolism

## Abstract

Oral anticoagulants (OACs) have been used to prevent stroke/systemic embolism (SE) among patients with non-valvular atrial fibrillation (NVAF). To evaluate baseline clinical characteristics, incidence rates of stroke/SE and hospitalization for bleeding, and OAC use among elderly patients with NVAF in the US by geographic region. Patients with NVAF were selected from the US Centers for Medicare & Medicaid Services claims database (01JAN2013-31DEC2016). Twelve months of health plan enrollment was required before and after the NVAF diagnosis to evaluate baseline characteristics and outcomes, respectively. Each patient was assigned to a 3-digit zip code based on their primary residence, and geographic variation was visualized using ArcGIS Pro software. Over 2.8 million patients with NVAF were identified. Large geographic variation was observed in clinical characteristics, stroke/SE, hospitalization for bleeding, and OAC use among patients across the US. The zip codes with the highest mean CHA_2_DS_2_–VASc scores and frequency of prior bleeding also had the highest incidence of stroke/SE and hospitalization for bleeding. Across 3-digit zip codes, 35–63% of patients were untreated. Overall, the incidence of stroke/SE and hospitalization for bleeding were higher and OAC treatment was less frequent in zip codes located in the Southern US. Baseline clinical characteristics, incidence rates of stroke/SE and hospitalization for bleeding, and OAC usage vary considerably by 3-digit zip code in the US. The additional granularity provided in this study may help clinicians to identify small regions with high-risk of stroke/SE and hospitalization for bleeding and low use of OAC that may benefit from targeted care strategies.

**Supplementary Information:**

The online version contains supplementary material available at 10.1007/s11239-023-02855-1.

## Highlights

- To examine unmet treatment needs among Medicare patients with non-valvular atrial fibrillation, geographic variations in baseline characteristics, incidence of stroke/systemic embolism and bleeding-related hospitalization, and oral anticoagulant use were assessed at the 3-digit zip code level.
- Overall, the South US region showed the lowest rates of oral anticoagulant treatment and the highest clinical risk scores and stroke/systemic embolism and bleeding incidence.
- The results may signal that hospital and clinical resources used to monitor warfarin are missing in these areas, preventing warfarin use in these high-risk areas.
- The higher level of granularity provided may allow public health programs, physicians, professional organizations, and health systems to focus on patient education campaigns, risk factor modification efforts, and provide care opportunities with much greater precision.
- These results may assist clinicians and other medical care providers in determining high-risk areas of the country.

## Introduction

Atrial fibrillation (AF), the most common chronic cardiac arrhythmia seen in clinical practice, is predicted to affect 12.1 million people in the United States by the year 2030 [1]. The incidence of stroke among patients with AF is nearly 5 times higher than that of the general population, resulting in significant morbidity and mortality in this patient population [2]. For several decades, warfarin has been the primary oral anticoagulant (OAC) used for stroke prevention among patients with non-valvular AF (NVAF), but in the last decade 4 direct oral anticoagulants (DOACs) have been approved in the United States and are often recommended over warfarin in guidelines due to their improved effectiveness and/or safety profiles [3, 4]. Nonetheless, all OAC therapies may carry a risk of major bleeding; therefore, it is important to monitor changes in bleeding risk as the AF treatment landscape continues to evolve [5].

Despite the burden of NVAF, little is known about the geographic variation in clinical outcomes of stroke/systemic embolism (SE). Some prior studies have shown that stroke/SE rates are higher in the southeastern US [6] corresponding to the “stroke belt,” a term coined by the National Heart, Lung, And Blood Institute to describe a region of the US associated with increased stroke mortality rates [7, 8]. In 2016, patients in the southeastern states comprising the stroke belt (Alabama, Arkansas, Georgia, Indiana, Kentucky, Louisiana, Mississippi, North and South Carolina, Tennessee, and Virginia) had a 27% higher age-adjusted stroke mortality rate compared to patients in the rest of the US [9, 10].

Although OACs are recommended for patients with NVAF and elevated stroke risk, OACs remain under-prescribed in the US—only about 50% of patients with NVAF and moderate or high risk of stroke/SE who are eligible for treatment per guidelines are prescribed an OAC [11, 12]. It is important to understand the geographic variation in the use of OACs in order to target certain geographic areas for treatment optimization. Hernandez et al. [13] examined the geographic variation in the use of OACs among patients with AF. The authors reported the likelihood of initiating any OAC therapy in each hospital referral region and found that the likelihood of initiation was highest in the Midwest and Northeast US [13]. Unfortunately the lack of regional granularity make it challenging to target quality improvement strategies to the areas with the highest need. No single study has evaluated the geographic variation of the incidence of stroke/SE and hospitalization for bleeding and the real-world OAC use patterns in the NVAF population.

To address these unmet needs, this retrospective analysis aimed to describe the geographic variation in the baseline risk of stroke/SE and history of bleeding, actual observed incidence of stroke/SE and hospitalization for bleeding, as well as variation in OAC use among US Medicare patients with NVAF by 3-digit zip code.

## Methods

This real-world retrospective analysis was performed in a database containing approximately 38 million US Centers for Medicare & Medicaid Services (CMS) beneficiaries between January 1, 2012 and December 31, 2017. The database contains medical and pharmacy claims from 100% national Medicare fee-for-service data, which includes hospital inpatient, outpatient, Medicare carrier, Part D pharmacy, skilled nursing facility, home health agency, and durable medical equipment files. The enrollment file contains demographic information on beneficiaries, including the zip code of their primary place of residence. Medical claims were obtained through the International Classification of Diseases, Ninth and Tenth Revision, Clinical Modification (ICD-9-CM/ICD-10-CM/ICD-10-PCS) diagnosis and procedure codes, as well as Health Care Common Procedure Coding System (HCPCS) and Current Procedural Terminology codes (CPT) (Supplemental Table 1). Drug prescriptions were identified through National Drug Codes (NDC).

Patients were selected if they had at least one inpatient or two outpatient claims with a diagnosis of AF in any position at least 7 days apart and within 365 days between January 1, 2013 and December 31, 2016 (patient identification period). The first AF claim during the identification period was designated as the index date. Patients were required to be ≥ 65 years of age on the index date. To evaluate baseline demographic and clinical characteristics as well as clinical outcomes, patients were required to have 12 months of continuous Medicare enrollment with medical and pharmacy benefits (Medicare Parts A, B, and D) prior to (baseline period) and after (follow-up period) the index date, including the index date. In order to evaluate NVAF, patients with rheumatic mitral valvular heart disease or valve replacement procedures during the 12-month baseline period were excluded from the final sample. Patients were followed from the index date until at least 12 months after the index date until Medicare disenrollment, death, or end of study period. Patients who died during the 12-month follow-up period were included in the study sample to avoid selection bias. 

Baseline demographic and clinical characteristics were evaluated during the 12-month baseline period at the 3-digit zip code level. The percentage of patients with a diagnosis code for bleeding (in any clinical setting) during the baseline period was calculated. In addition, mean CHA_2_DS_2_–VASc and modified HAS-BLED scores were calculated for each 3-digit zip code. The CHA_2_DS_2_–VASc score, which evaluates the risk of stroke among patients with NVAF on a scale from 0 to 9 was calculated based on the following characteristics: congestive heart failure; hypertension; age ≥ 75 years; diabetes mellitus; history of stroke, transient ischemic attack, or thromboembolism; vascular disease; age 65–74 years; and sex category[14]. The modified HAS-BLED score, which estimates the 1-year risk of major bleeding among patients with AF on a scale of 0–8, was calculated based on the following 6 characteristics: hypertension, abnormal kidney and/or liver function, stroke, bleeding, elderly (age > 65), and alcohol/drug therapy. The labile international normalized ratio used in the standard HAS-BLED was not available in the database [15]. In our study population, the minimum score was 1 as all patients were ≥ 65 years of age. 

Stroke/SE incidence and hospitalization for bleeding incidence were evaluated during the at least 12-month follow-up period (including the index date AF diagnosis). Stroke/SE cases were based on hospitalizations with stroke/SE as the principal diagnosis. Hospitalization for bleeding was defined as hospitalization with a bleeding event as the principal diagnosis which is consistent with the major bleeding definition as previously published [16]. Incidence rates (per 100 person-years) were calculated as the number of patients with a case divided by the total time at risk for each patient during the follow-up period. 

Prevalent OAC treatment usage during the at least 12-month follow-up period, including the index date, was evaluated among those patients with a CHA_2_DS_2_–VASc ≥ 2 based on the clinical recommendations at the time of study initiation. This was chosen to reflect the guidance provided by professional societies for OAC treatment in patients with NVAF during data availability. Male and female patients with NVAF were recommended to receive OAC for CHA_2_DS_2_–VASc ≥ 2 prior to the update in 2019 [3]. Patients who were prescribed warfarin or a DOAC were identified and categorized according to the first OAC prescription filled on or after the index date.

Data analysis was performed using statistical software SAS version 9.4 (SAS Institute Inc., Cary, NC, USA). Based on their primary residence, each patient was assigned to one of the 929 US 3-digit zip codes. Data were uploaded to ArcGIS Pro software in order to visualize the observed geographic variation in baseline CHA_2_DS_2_-VASc and modified HAS-BLED scores, the proportion of baseline bleeds, the follow-up incidence rates of stroke/SE and hospitalization for bleeding, and OAC treatment usage [17]. The data in each figure were displayed in quintiles.

Since this study did not involve the collection, use, or transmittal of individually identifiable data, it was exempt from Institutional Review Board review. Both the datasets and the security of the offices where analysis was completed (and where the datasets are kept) meet the requirements of the Health Insurance Portability and Accountability Act of 1996.

## Results

After applying the inclusion and exclusion criteria, 2,828,416 patients with NVAF were included in the study sample; 2,756,097 (97.4%) had a CHA_2_DS_2_–VASc score ≥ 2 during the baseline period (Supplemental Fig. 1). The mean age in the study sample was 79.6 years, and a majority of patients were female (55.2%) and White (89.0%). Additional demographic and clinical characteristics are shown in Table 1.

**Table 1:** Demographics, clinical characteristics, and outcomes of medicare patients with NVAF

	Patients with NVAF	
	N/Mean	%/SD
Sample Size	2,828,416	
Age, years	79.6	8.2
65–74	877,366	31.0%
75–79	550,926	19.5%
≥ 80	1,400,124	49.5%
Sex		
Male	1,266,651	44.8%
Female	1,561,765	55.2%
Race/Ethnicity		
White	2,517,205	89.0%
African American	175,049	6.2%
Hispanic	44,387	1.6%
Other	91,775	3.2%
Baseline^a^		
Deyo-Charlson Comorbidity Index (CCI)	3.0	2.9
CHA_2_DS_2_ –VASc Score	4.4	1.8
HAS-BLED Score	3.1	1.3
Stroke/SE Incidence (per 100-py)	1.9	
Hospitalization for Bleeding Incidence (per 100-py)	3.7	
CHA_2_DS_2_ –VASc ≥ 2	2,756,097	97.4%
Untreated^b^	1,339,516	48.6%
Treated^b^	1,416,581	51.4%
Warfarin	854,001	31.0%
DOAC	562,580	20.4%

DOAC: direct oral anticoagulant, NVAF: non-valvular atrial fibrillation, SD: standard deviation

^a^Comorbidities were defined using diagnosis codes in any claim position

^b^Untreated, treated, warfarin, and DOAC proportions are subsets of the patient population with a baseline CHA_2_DS_2_ –VASc Score ≥ 2 (n = 2,756,097)

**Fig. 1:**
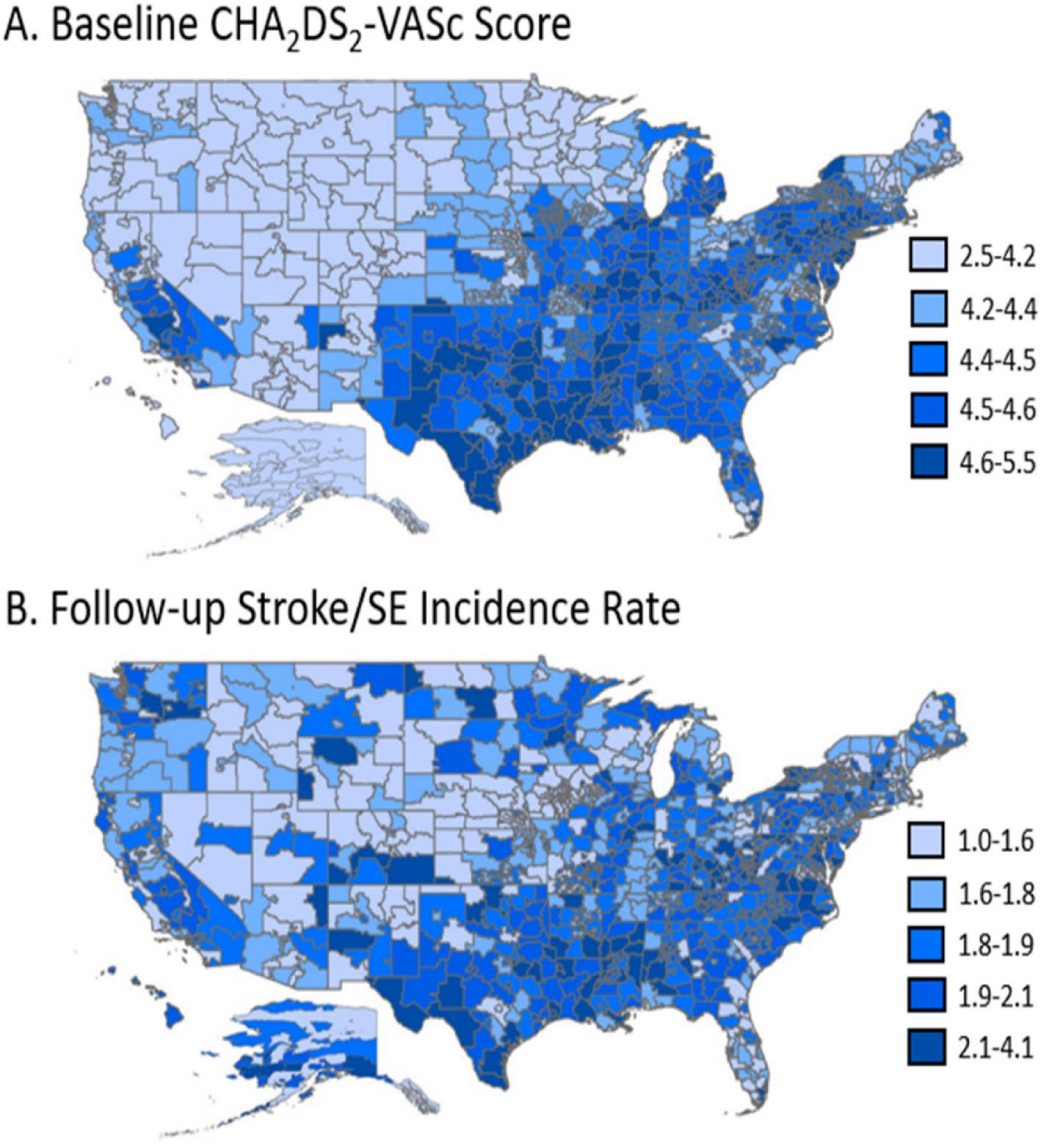
Geographic Variation of Baseline CHA_2_DS_2_-VASc Score (**A**) and Follow-up Stroke/SE Incidence Rate (**B**) Among Medicare Patients with NVAF from 2013–2016. Baseline CHA_2_DS_2_-VASc score and follow-up stroke/SE incidence rates were measured among all patients with NVAF by 3-digit zip code (N = 2,828,416). Incidence rates were measured per 100 person-years. Regions with higher risk scores were also the areas of higher stroke/SE incidence

Similar geographic variation patterns were observed with the mean baseline CHA_2_DS_2_ –VASc stroke risk score and the follow-up incidence of stroke/SE, with higher risk scores and higher incidence rates occurring in the Southern region of the US. The mean CHA_2_DS_2_–VASc score among all patients with NVAF was 4.4 and ranged from 2.5 to 5.5 across all 3-digit zip codes (Fig. 1). The overall incidence of stroke/SE during the follow up period was 1.9 cases per 100 person-years and ranged from 1.0 to 4.1 cases per 100 person-years across all 3-digit zip codes.

**Fig. 2:**
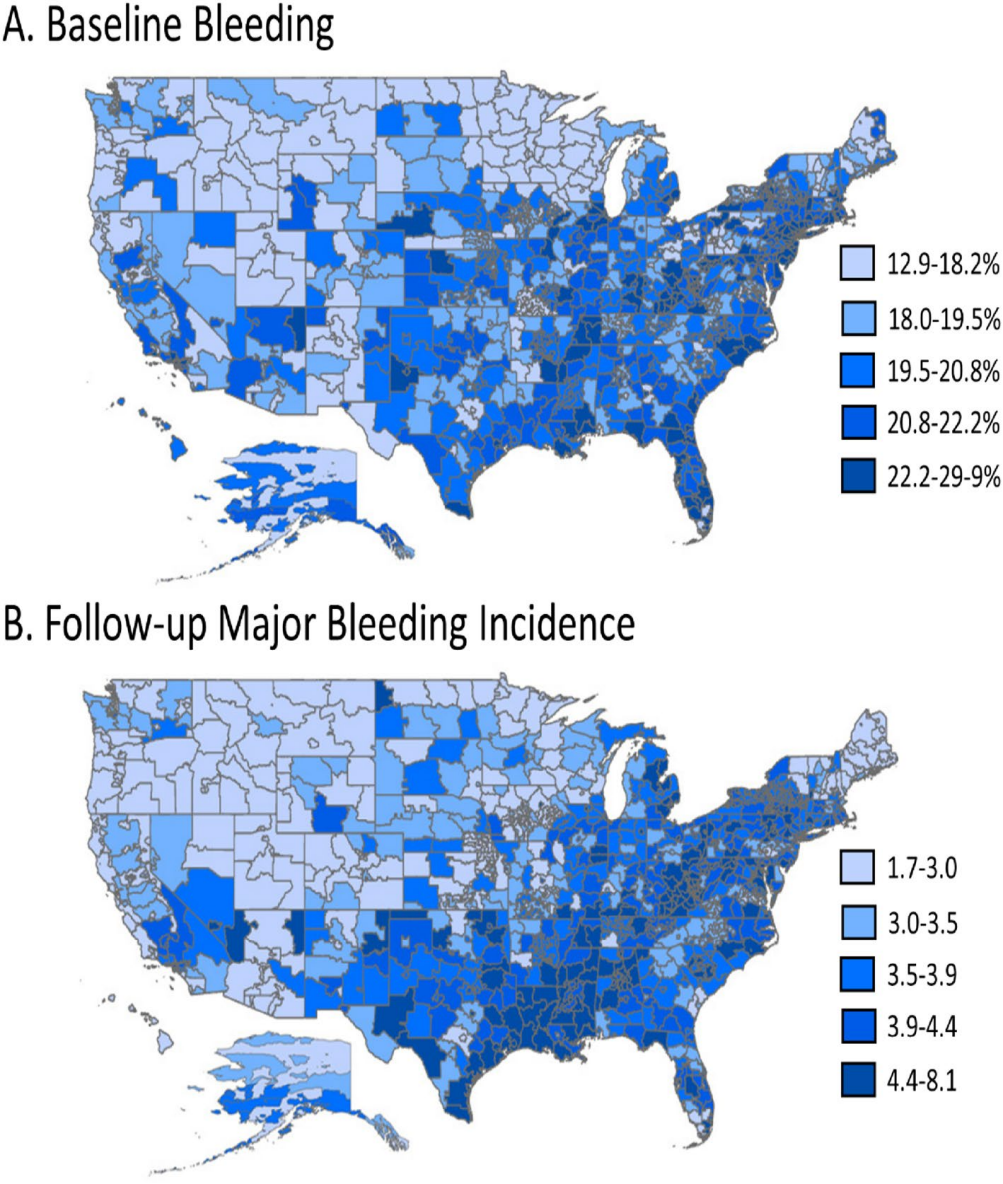
Geographic Variation of Baseline Bleeds (**A**) and Follow-up Hospitalization for Bleeding Incidence Rate (**B**) Among Medicare Patients with NVAF from 2013–2016. Prevalence of baseline bleeds and follow-up hospitalization for bleeding incidence rates were measured among all patients with NVAF by 3-digit zip code (N = 2,828,416). Incidence rates were measured per 100 person-years. Hospitalization for bleeding was assessed after the NVAF diagnosis regardless of OAC treatment. Regions with higher prevalence of baseline bleeds were similar to the areas of higher incidence of hospitalizations for bleeding

Similar variation was observed between prevalence of baseline bleeds and the incidence of hospitalization for bleeding, with higher prevalence and higher incidence rates observed in the Southern and Eastern United States relative to other regions. Approximately 20% of all patients experienced a bleeding event in the baseline period, ranging from 12.9 to 29.9% across all 3-digit zip codes (Fig. 2). In addition, the mean HAS-BLED score was 3.1 and had a similar trend in regional differences as baseline bleeding (Supplemental Fig. 2). The overall incidence rate of hospitalization for bleeding among all patients (treated and untreated with OACs) was 3.7 cases per 100 person-years, ranging from 1.7 to 8.1 cases per 100 person-years.

**Fig. 3:**
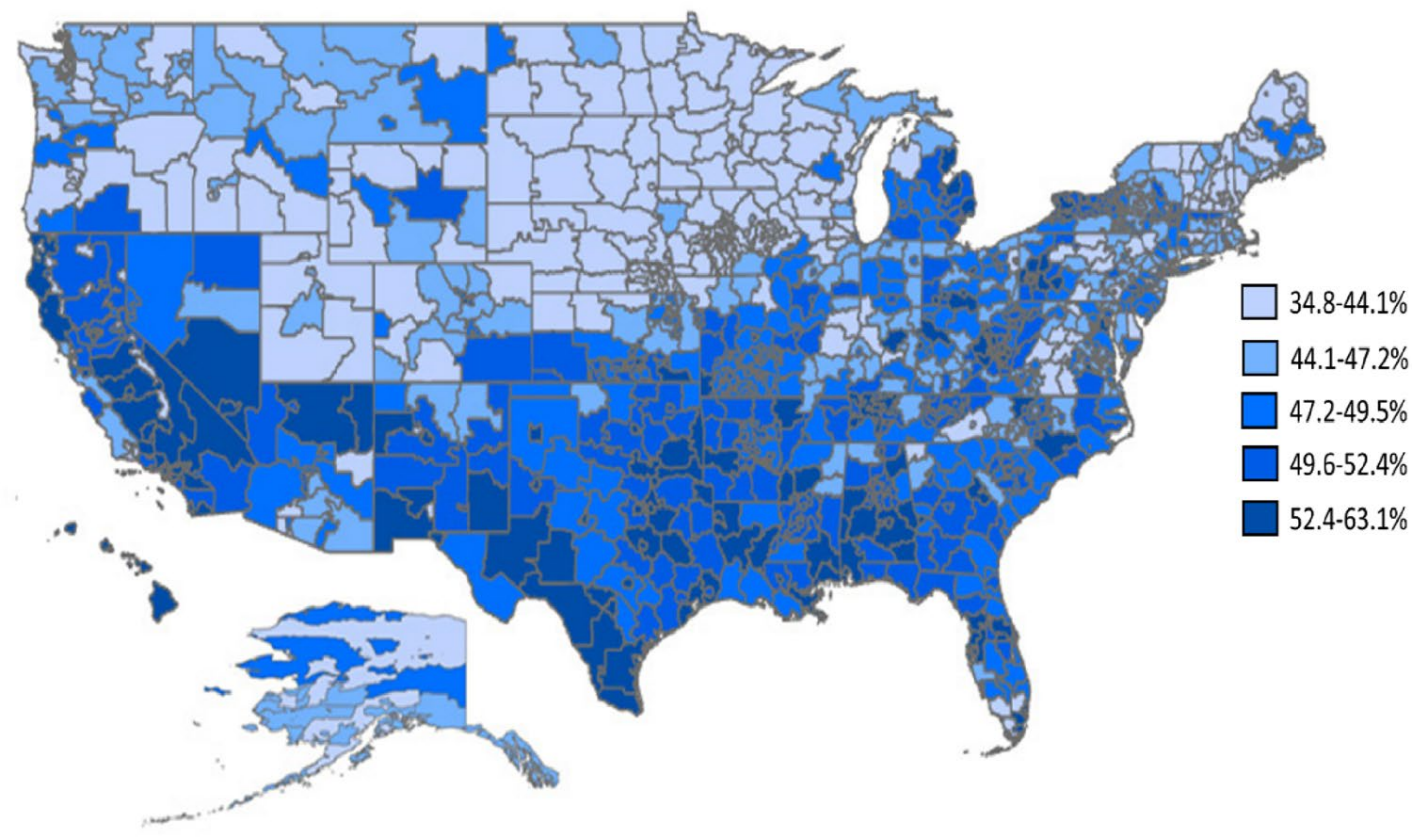
Geographic Variation of OAC-untreated Patients among Medicare Patients with NVAF and baseline CHA_2_DS_2_ –VASc score ≥ 2 from 2013 to 2016. OAC-untreated patients in the follow-up period were evaluated among those patients with NVAF and a CHA_2_DS_2_-VASc ≥ 2 during the baseline period (N = 2,756,097). Untreated patients most commonly resided in the Southern region of the US

Among patients with NVAF and a CHA_2_DS_2_–VASc score ≥ 2 (N = 2,756,097), 1,339,516 (48.6%) were not prescribed an OAC during the follow-up period. Figure 3 shows the geographic variation of OAC-untreated patients by 3-digit zip code. The percentage of patients with NVAF and CHA_2_DS_2_ –VASc score ≥ 2 who were untreated with an OAC ranged from 34.8 to 63.1% across 3-digit zip codes, with the highest untreated populations generally residing in the Southern region of the US.

**Fig. 4:**
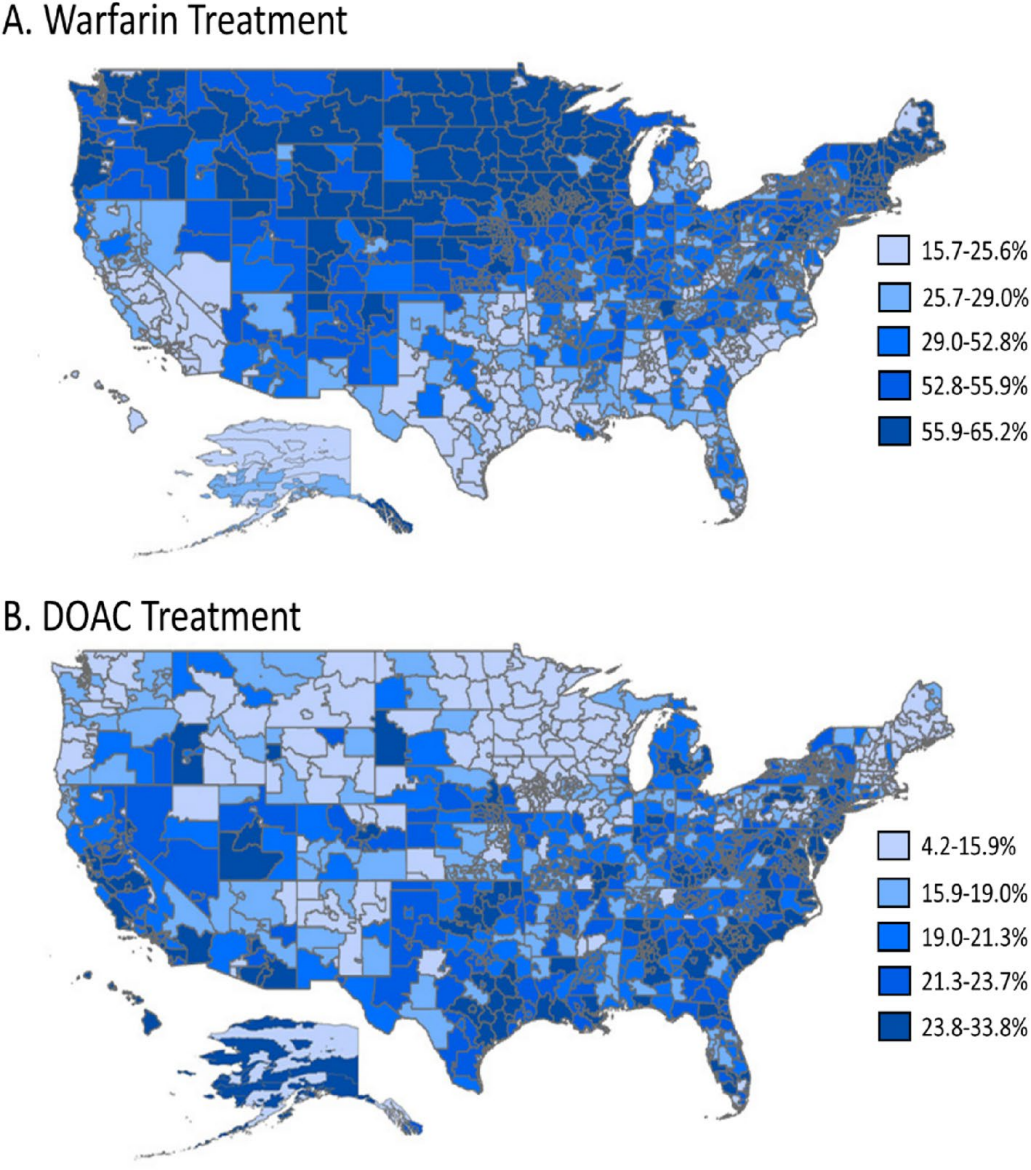
Geographic Variation of Warfarin Treatment (**A**) and DOAC Treatment (**B**) During the Follow-up Period Among Medicare Patients with NVAF and baseline CHA_2_DS_2_ –VASc score ≥ 2 from 2013 to 2016. OAC treatment in the follow-up period was measured among those patients with NVAF and a CHA_2_DS_2_-VASc ≥ 2 during the baseline period (N = 2,756,097). Patients were assigned to the cohort according to the first prescription received in the follow-up period. Warfarin treatment was most common in the Midwest regions while DOAC treatment was most common in the Southern region of the US

Among patients with NVAF and a CHA_2_DS_2_–VASc score ≥ 2 (n = N = 2,756,097), 854,001 (31.0%) and 562,580 (20.4%) were treated with warfarin or a DOAC, respectively (51.4% [1] were OAC treated). The geographic variation in warfarin and DOAC treatment is displayed in Fig. 4. DOAC was the OAC of choice in 4.2–33.8% of patients across 3-digit zip codes. Lower DOAC treatment rates were seen in the Midwest region while those patients more likely to be DOAC-treated resided in 3-digit zip codes located in the Southern region of the US. The geographic variation in patients with NVAF and a CHA_2_DS_2_–VASc score ≥ 2 who were treated with either warfarin or DOACs is shown in Supplemental Fig. 3.

## Discussion

This study evaluated the real-world geographic variation in incidence rates of stroke/SE and hospitalization for bleeding, and OAC treatment among patients with NVAF in the US at the 3-digit zip code level. Among all study patients with NVAF, we found that the 3-digit zip codes with higher mean CHA_2_DS_2_–VASc stroke risk scores–located primarily in the Southern region–were the same 3-digit zip codes associated with higher stroke incidence rates. A similar pattern was observed for the prevalence of baseline bleeds and mean baseline HAS-BLED score and the incidence rate of hospitalization for bleeding across the US. Together, these data verify the potential usefulness of these scores for identifying higher risk patients with NVAF. 

These results improve the granularity of data provided in previous studies that have evaluated the geographic variation in these outcomes associated with NVAF. Reporting outcomes at the 3-digit zip code level may more effectively aid interpretation compared to studies that have reported outcomes on a state or region-level. The 3-digit zip code breaks up each state into multiple areas to observe variations within states instead of summarizing at the state or regional level. For example, in this study there is clear evidence of higher stroke/SE rates in the Southern US, corresponding to the stroke belt. These results differ from those reported by Claxton et al. who did not report such a pattern [18]. While the differences may be due to the databases used in each study, it is also likely that the regional variation is better represented and visualized at this level where trends for entire regions are less likely to be influenced by individual cities or metropolitan areas. While our results are consistent with previous studies that have reported higher stroke/SE rates in the southeastern US, the higher level of granularity provided in our study may allow public health programs, physicians, professional organizations, and health systems to focus patient education campaigns, risk factor modification efforts, and provide care opportunities with much greater precision [7, 9, 19].

Apart from the presence of a stroke belt as hypothesized by previous literature, other trends were also observed between outcomes across the US. The 3-digit zip codes with the highest stroke risk and stroke/SE events generally corresponded to the 3-digit zip codes with lower usage of OAC treatment. Approximately half (35–63% across 3-digit zip codes) of all patients with NVAF and CHA_2_DS_2_–VASc score ≥ 2 were not prescribed an OAC, which is consistent with the findings reported by Hernandez et al. While not all patients with NVAF and CHA_2_DS_2_–VASc score ≥ 2 are eligible for OAC treatment, due to high risk of bleeding, history of prior bleeding, or prior surgical or minimally invasive procedures for stroke risk reduction, it is clear from our data that opportunities still exist to improve stroke risk reduction in this vulnerable population. 

Similar to the findings of Hernandez et al., the areas with lower overall OAC usage corresponded to the areas with the highest percentage of DOAC treatment (Southern region), suggesting that DOACs are the preferred treatment among patients with NVAF who are eligible for treatment with OAC in these areas. Since DOACs have been found to have greater convenience (no need for routine INR monitoring and few dietary restrictions and DDIs) and improved safety and effectiveness in patients with NVAF but incur higher costs compared to vitamin K antagonist treatment alternatives, this indicates that in patients who receive treatment, favorable clinical outcomes are being chosen over affordability in these areas with a concentration of high-risk patients [20]. Alternatively, this may be a signal that hospital and clinic resources needed to use and monitor warfarin are missing in these areas, preventing to some degree the use of warfarin treatment in these high stroke/SE risk areas. Underutilization may then be driven by cost in these areas, where the socioeconomic status may be lower, however all patients in our analysis had Medicare part D (prescription) benefits. Overall, the low OAC use in the south may lend itself to programs and other factors to improve use of OACs which could include reduced barriers to treatment (reduced cost, increased availability) in addition to educational interventions as done by Vinereanu et al. [21].

Despite its contribution in providing real-world evidence, this study is subject to limitations. First, an inherent limitation to retrospective claims database analysis is that the medical claims are collected for administrative purposes and not research, meaning that the presence of a claim for a filled prescription does not necessarily indicate that the medication was taken as prescribed. Similarly, medications filled in a hospital or over-the-counter cannot be observed in the database. Second, since this study assigned each patient to the OAC cohort based on the first prescription received during the follow-up period, treatment switching and discontinuation were not captured. Third, stroke/SE and hospitalization for bleeding outcome events were evaluated any time after the NVAF diagnosis, regardless of treatment, so association between a hospitalization for bleeding and OAC or DOAC treatment could not be established. Fourth, this study was performed in a Medicare population over the age of 65 with prescription drug coverage; as such, their risk of stroke/SE and hospitalization for bleeding was high. Our results may not apply to younger patients, those with other forms of insurance, and those outside of the US. Similar research like this should be replicated in other parts of the world. Fifth, the clinical guidelines for OAC treatment among patients with NVAF have changed since the time of study initiation. We reported treatment among patients with NVAF and a CHA_2_DS_2_–VASc score ≥ 2, which matched the clinical guidelines during the study period; however, current guidelines recommend treatment for males with a CHA_2_DS_2_–VASc score ≥ 2 and females with a CHA_2_DS_2_–VASc score ≥ 3 [22]. Sixth, as the results were reported at the finest level of granularity available, certain patient values represented visually in different quintiles may not have a clinically meaningful difference. Seventh, diagnosis of NVAF relied on ICD-9 and ICD-10 diagnosis codes which do not distinguish between chronic and acute reversable NVAF. Finally, additional factors might have influenced geographic variation and low rates of OAC use and were not evaluated in this study, including socio-demographic characteristics, hospital characteristics, and access to resources for the management of patients with NVAF. These may represent areas of future research, particularly the predictors of low rates of OAC use.

## Conclusion

To summarize, this study described the geographic variation in baseline characteristics, incidence rates of stroke/SE and hospitalization for bleeding, and the use of OACs among Medicare patients with NVAF at the 3-digit zip code level. Areas with the highest clinical risk scores corresponded to the areas with the highest incidence rates of stroke/SE and bleeding, which were mainly located in the Southern region of the US where the lowest rates of overall OAC treatment were observed. DOAC treatment was most common in the Southern region of the US while warfarin was most common in the Midwest region. These results provide a level of granularity which may assist clinicians and other medical care providers in determining high-risk areas of the country which may have unmet treatment needs.

### Supplementary Information

Below is the link to the electronic supplementary material.

Supplementary file1 (DOCX 1895 KB)

Supplementary file2 (TIF 575 KB)

Supplementary file3 (TIF 1168 KB)

Supplementary file4 (TIF 1473 KB)

## Data Availability

The raw insurance claims data used for this study originate from Medicare data, which are available from the Centers for Medicare and Medicaid through ResDAC (https://www.resdac.org/). Other researchers could access the data through ResDAC, and the inclusion criteria specified in the Methods section would allow them to identify the same cohort of patients we used for these analyses.

## Abbreviations

AF: Atrial fibrillation
CMS: Centers for Medicare & Medicaid Services
DOAC: Direct oral anticoagulant
NVAF: Non-valvular atrial fibrillation
OAC: Oral anticoagulant
SE: Systemic embolism
