# Supplementary material for: Geographic variation in clinical outcomes and anticoagulation among medicare beneficiaries with non-valvular atrial fibrillation
Source: J Thromb Thrombolysis. 2023 Aug 2;56(4):626–34. doi: 10.1007/s11239-023-02855-1 (PMC10550860; doi:10.1007/s11239-023-02855-1)
Supplement: Supplementary file 1 — Supplementary file1 (DOCX 1895 KB) [file 11239_2023_2855_MOESM1_ESM.docx]

**Supplemental Figure 1. Study Selection Criteria**

**
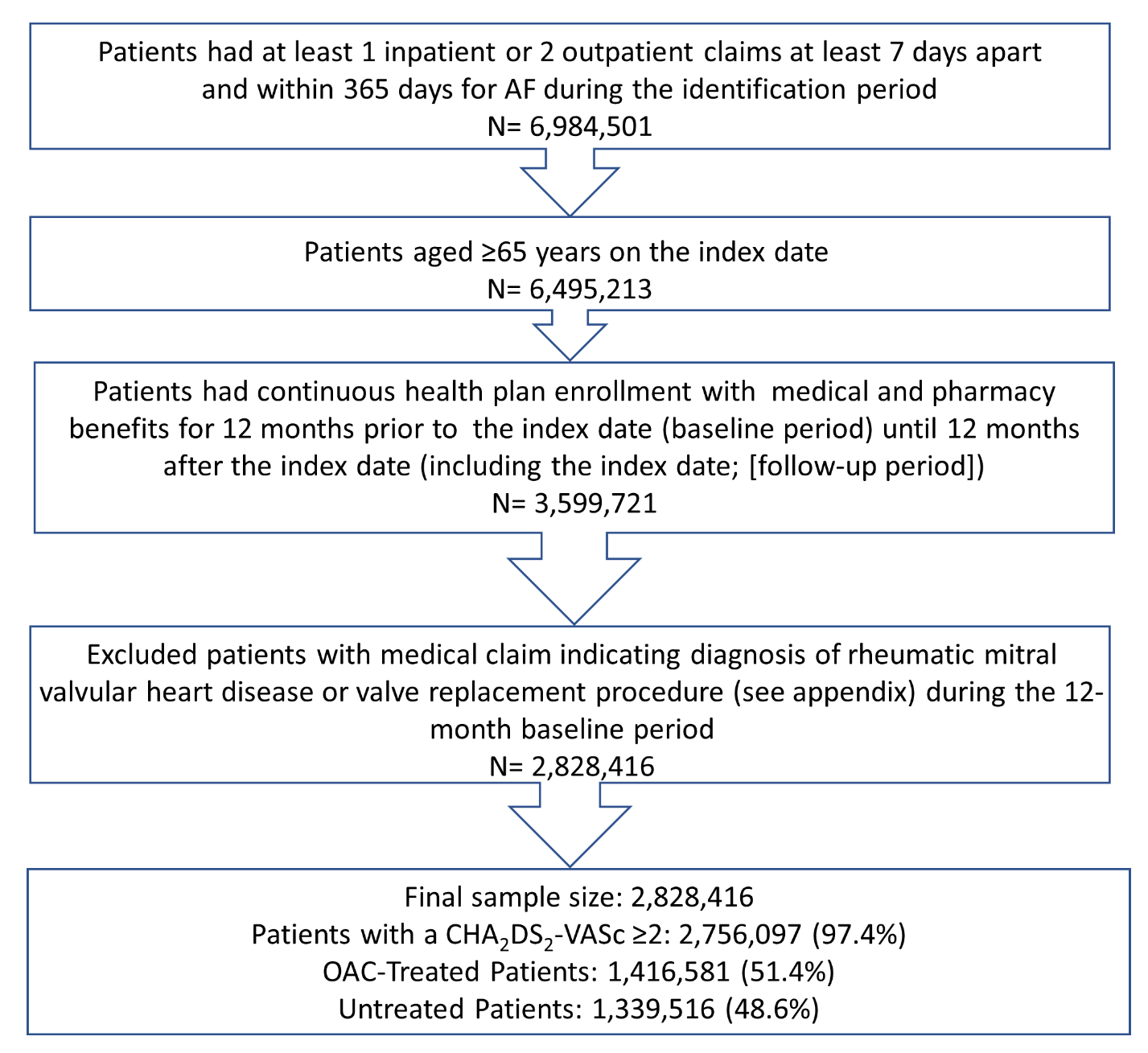
**

**Supplemental Figure 2. Geographic Variation of baseline modified HAS-BLED score among Medicare Beneficiaries with NVAF from 2013-2016**

**
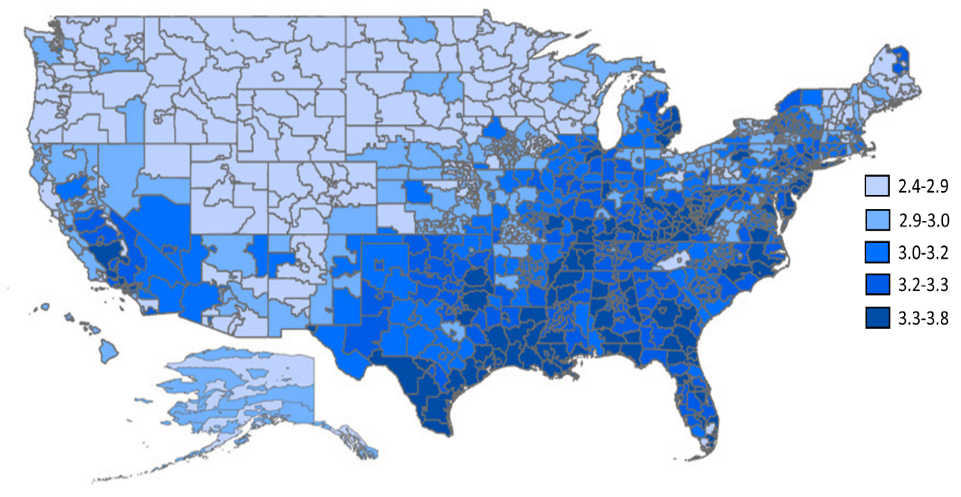
**

**N=**2,828,416 patients with NVAF were included in the study sample.

**Supplemental Figure 3. Geographic Variation of OAC Treatment among Medicare Beneficiaries with NVAF with baseline CHA2DS2 –VASc score ≥ 2 from 2013-2016**

**
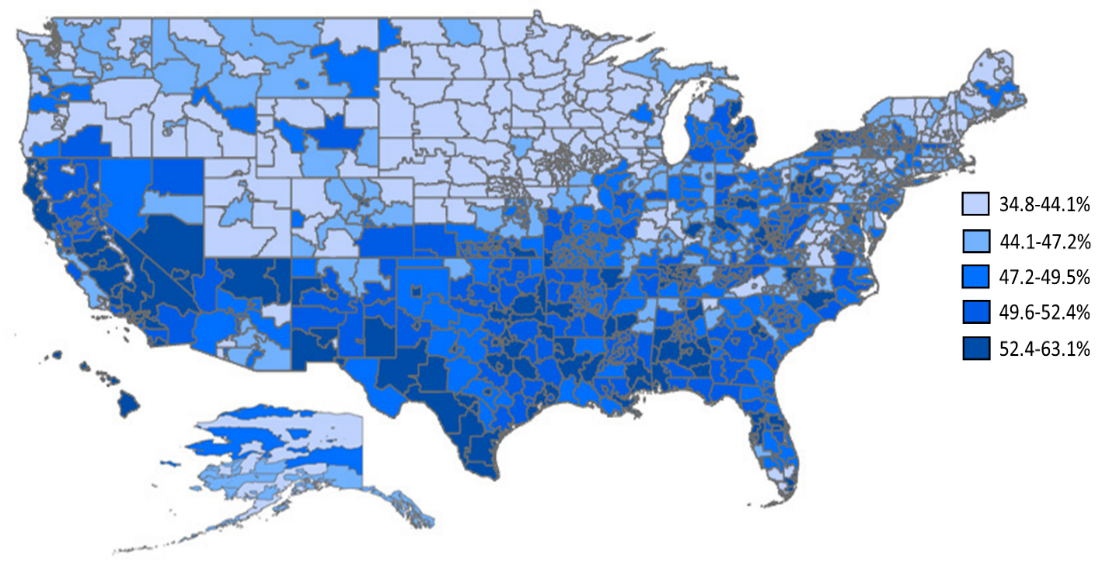
**

N=2,756,097 patients had a CHA2DS2 –VASc score ≥ 2.

**Supplemental Table 1. Diagnosis and Procedure Codes for Patient Selection Criteria**

| **Diagnosis** | **ICD-9-CM Codes** | **ICD-10-CM/PCS Codes** |
| --- | --- | --- |
| Atrial Fibrillation (AF) | 427.31 | I480.x, I482.x, I481.x, I483.x, I484.x, I4891.x, I4892.x |
| Valvular Heart Disease | 394.0, 394.1, 394.2, 394.9, 396.0, 396.1, 396.8, 396.9, 424.0, 745.xx | I050.x, I051.x, I052.x, I058.x, I059.x, I080.x, I088.x, I089.x, I340.x, I341.x, I342.x, I348.x, I349.x, Q213.x, Z952.x, Z953.x, Z954.x |
| Valve Replacement Procedure | 35.05-35.09, 35.20-35.28, 35.97 | 02RF07Z, 02RF08Z, 02RF0JZ, 02RF0KZ, 02RF37H, 02RF37Z, 02RF38H, 02RF38Z, 02RF3JH, 02RF3JZ, 02RF3KH, 02RF3KZ, 02RF47Z, 02RF48Z, 02RF4JZ, 02RF4KZ, 02RG07Z, 02RG08Z, 02RG0JZ, 02RG0KZ, 02RG37H, 02RG37Z, 02RG38H, 02RG38Z, 02RG3JH, 02RG3JZ, 02RG3KH, 02RG3KZ, 02RG47Z, 02RG48Z, 02RG4JZ, 02RG4KZ, 02RH07Z, 02RH08Z, 02RH0JZ, 02RH0KZ, 02RH37H, 02RH37Z, 02RH38H, 02RH38Z, 02RH3JH, 02RH3JZ, 02RH3KH, 02RH3KZ, 02RH47Z, 02RH48Z, 02RH4JZ, 02RH4KZ, 02RJ07Z, 02RJ08Z, 02RJ0JZ, 02RJ0KZ, 02RJ47Z, 02RJ48Z, 02RJ4JZ, 02RJ4KZ, 02UG3JZ, X2RF032, X2RF332, X2RF432 |
