# Supplementary figures and images for: Geographic variation in clinical outcomes and anticoagulation among medicare beneficiaries with non-valvular atrial fibrillation
Source: J Thromb Thrombolysis. 2023 Aug 2;56(4):626–34. doi: 10.1007/s11239-023-02855-1 (PMC10550860; doi:10.1007/s11239-023-02855-1)

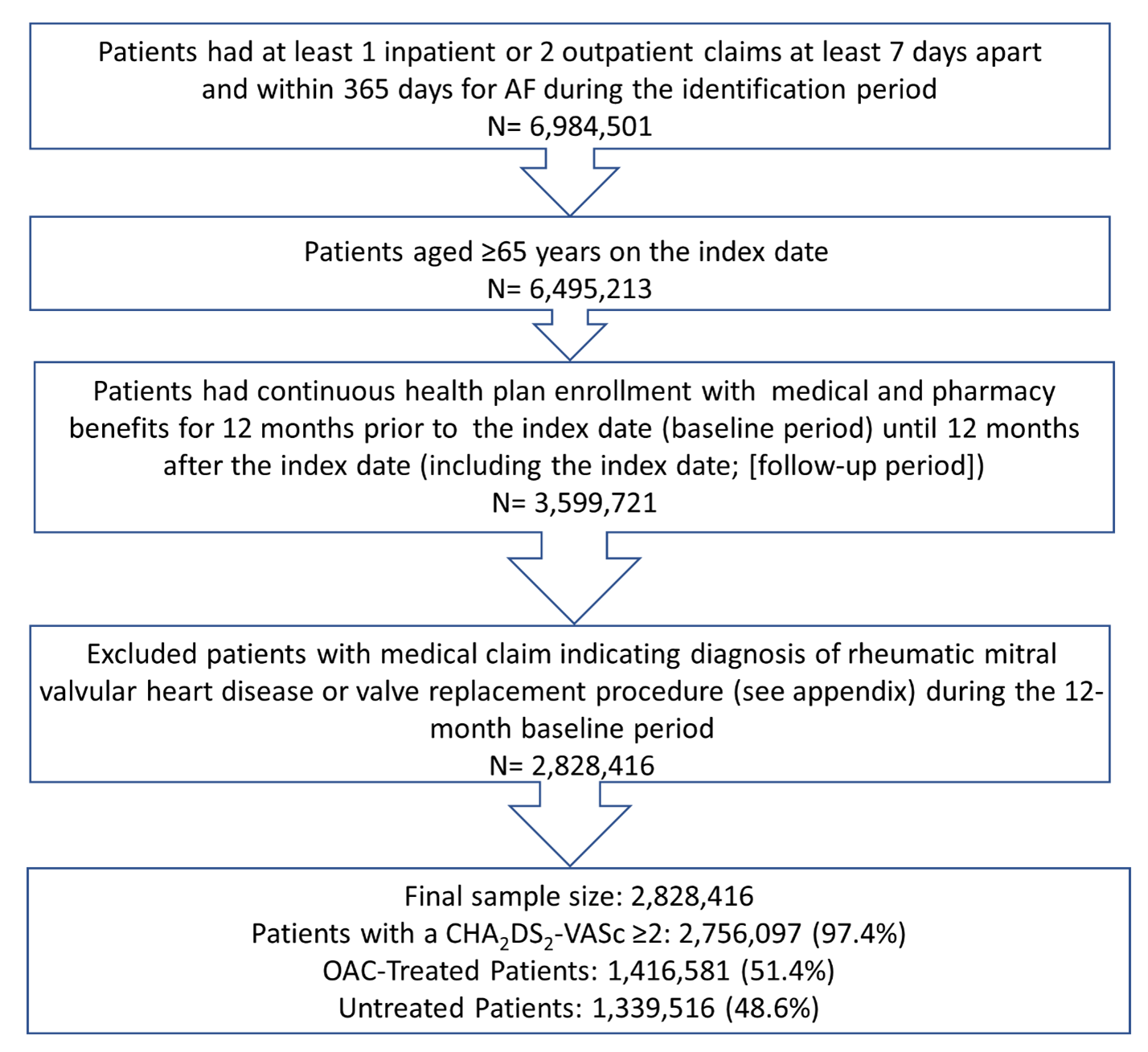

Supplement: Supplementary file 2 — Supplementary file2 (TIF 575 KB) [file 11239_2023_2855_MOESM2_ESM.tif]

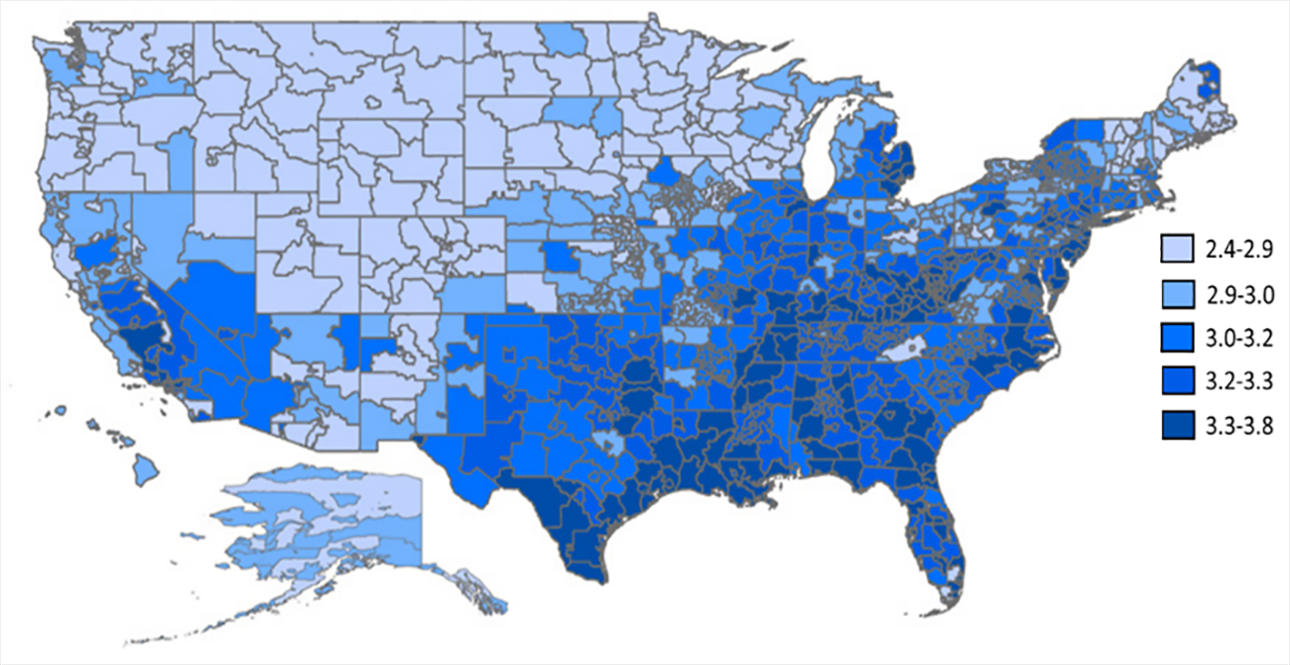

Supplement: Supplementary file 3 — Supplementary file3 (TIF 1168 KB) [file 11239_2023_2855_MOESM3_ESM.tif]

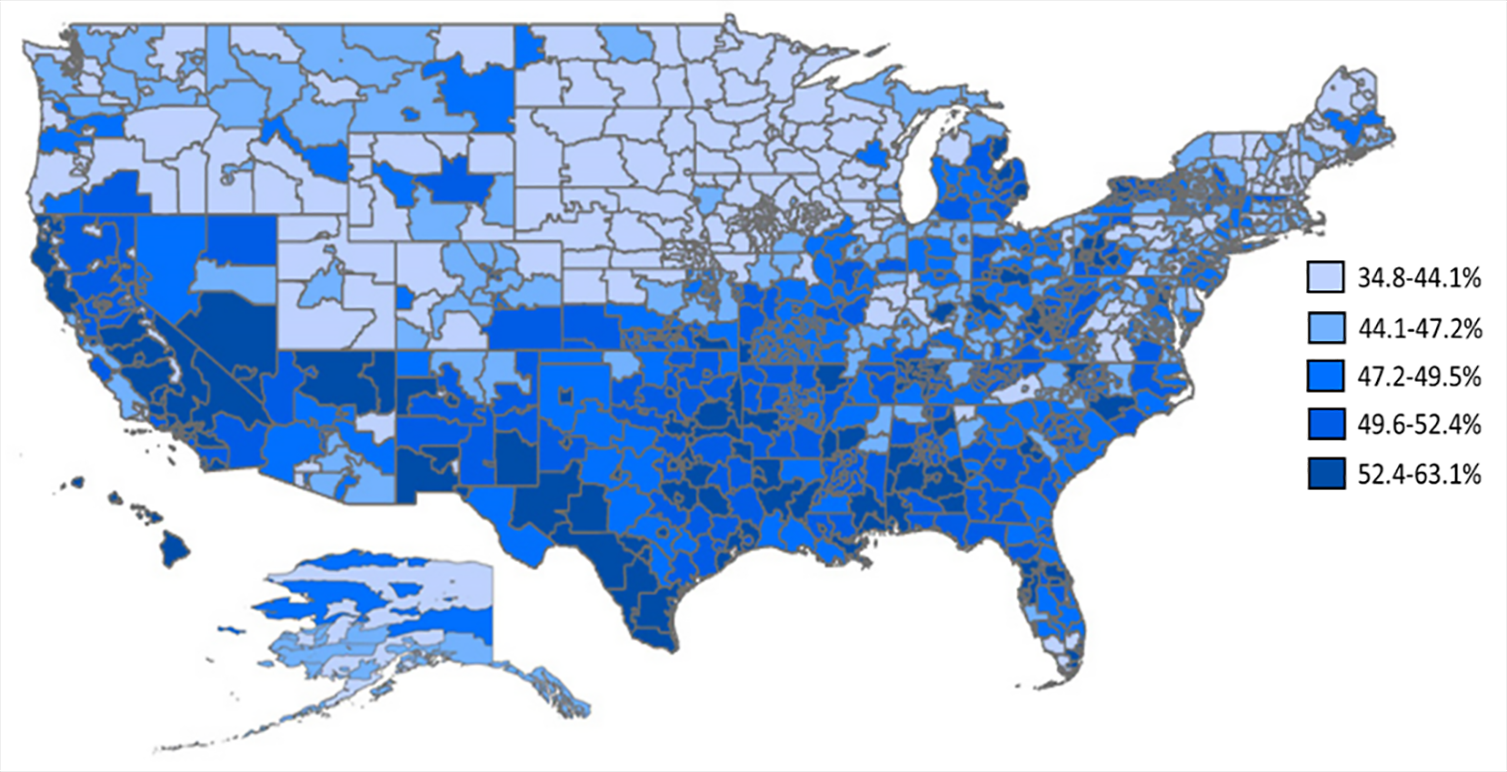

Supplement: Supplementary file 4 — Supplementary file4 (TIF 1473 KB) [file 11239_2023_2855_MOESM4_ESM.tif]
